# Pembrolizumab Combined With Neoadjuvant Chemotherapy Versus Neoadjuvant Chemoradiotherapy Followed by Surgery for Locally Advanced Oesophageal Squamous Cell Carcinoma: Protocol for a Multicentre, Prospective, Randomized-Controlled, Phase III Clinical Study (Keystone-002)

**DOI:** 10.3389/fonc.2022.831345

**Published:** 2022-03-31

**Authors:** Xiaobin Shang, Wencheng Zhang, Gang Zhao, Fei Liang, Chen Zhang, Jie Yue, Xiaofeng Duan, Zhao Ma, Chuangui Chen, Qingsong Pang, Weihong Zhang, Liang Liu, Xiubao Ren, Bin Meng, Peng Zhang, Yegang Ma, Lin Zhang, Hecheng Li, Xiaozheng Kang, Yin Li, Hongjing Jiang

**Affiliations:** ^1^Department of Minimally Invasive Esophageal Surgery, Key Laboratory of Cancer Prevention and Therapy, National Clinical Research Center for Cancer, Tianjin Medical University Cancer Institute and Hospital, Tianjin, China; ^2^Department of Radiation Oncology, Key Laboratory of Cancer Prevention and Therapy, National Clinical Research Center for Cancer, Tianjin Medical University Cancer Institute and Hospital, Tianjin, China; ^3^Department of Pathology, Key Laboratory of Cancer Prevention and Therapy, National Clinical Research Center for Cancer, Tianjin Medical University Cancer Institute and Hospital, Tianjin, China; ^4^Department of Biostatistics, Zhongshan Hospital, Fudan University, Shanghai, China; ^5^Department of Immunology, Key Laboratory of Cancer Prevention and Therapy, National Clinical Research Center for Cancer, Tianjin Medical University Cancer Institute and Hospital, Tianjin, China; ^6^Department of Thoracic Surgery, Tianjin Medical University General Hospital, Tianjin, China; ^7^Department of Thoracic Surgery, Liaoning Cancer Hospital, Shenyang, China; ^8^Department of Thoracic Surgery, Shandong Provincial Hospital Affiliated to Shandong First Medical University, Jinan, China; ^9^Department of Thoracic Surgery, Ruijin Hospital, Shanghai Jiao Tong University School of Medicine, Shanghai, China; ^10^Department of Thoracic Surgical Oncology, Chinese Academy of Medical Sciences and Peking Union Medical College, National Cancer Center/Cancer Hospital, Beijing, China

**Keywords:** oesophageal cancer (EC), neoadjuvant therapy, immunotherapy, efficacy, event-free survival (EFS)

## Abstract

**Background:**

To compare the efficacy and safety of pembrolizumab combined with neoadjuvant chemotherapy (neoCT) versus neoadjuvant chemoradiotherapy (neoCRT) followed by surgery for locally advanced resectable oesophageal squamous cell carcinoma (ESCC).

**Methods:**

This study is a multicentre, prospective, randomized-controlled, phase III clinical study. Eligible ESCC (staging: cT1N2M0 or cT2-3N0-2M0 (stage II/III, high-risk lesions in T2N0M0)) patients will be randomly assigned to either the experimental group (pembrolizumab with neoCT, n = 228) or the control group (neoCRT, n = 114) at a ratio of 2:1. Within 4–6 weeks after preoperative therapy, the McKeown procedure will be performed. Patients in the experimental group will also receive pembrolizumab alone as adjuvant therapy after surgery until 1 year or until the radiographically confirmed PD or other condition indicated for premature termination is observed. The primary endpoint is event-free survival (EFS). The secondary endpoints are 1-, 3-, and 5-year overall survival (OS) and disease-free survival (DFS), short-term outcomes, and quality of life.

**Discussion:**

This is the first prospectively randomized controlled trial designed to compare pembrolizumab plus chemotherapy and chemoradiotherapy as neoadjuvant therapy for resectable ESCC. According to our hypothesis, preoperative pembrolizumab combined with chemotherapy will result in a better tumour response and prolong the survival of patients, with acceptable toxicity. This study started in December 2021, and the enrolment time is estimated to be 2 years.

**Trial Registration:**

This prospective study has been registered at ClinicalTrials.gov (NCT04807673), March 2021.

## Background

Oesophageal cancer (EC) ranks as the seventh most common cancer and the sixth most common cause of cancer-related death worldwide (1). Approximately 50% of EC cases occur in China, of which more than 90% are oesophageal squamous cell cancer (ESCC) (2). Oesophagectomy is the primary treatment for locally advanced resectable EC, which has a 5-year survival rate of only approximately 20%, and the recurrence or metastasis rate is high with surgery alone (3). The National Comprehensive Cancer Network (NCCN) guideline and Chinese Society of Clinical Oncology (CSCO) guideline both recommend neoCRT followed by oesophagectomy as the standard treatment for locally advanced ESCC, with an over 40% of pathological complete response (pCR) rate (4, 5). Nevertheless, neoCRT that has been shown to increase difficulty of surgery and perioperative complications needs to be carried out at high-volume and experienced centres. Therefore, it is necessary to seek new treatment strategies to improve the prognosis of ESCC and ensure acceptable safety in treatment approaches.

Pembrolizumab is a monoclonal antibody (mAb) against humanized immune globulin G4 (IgG4), which specifically binds to programmed cell death receptor 1 (PD-1). To date, a series of high-level evidence-based medical studies have confirmed the good efficacy of pembrolizumab for advanced EC. In the phase III KEYNOTE-181study (6), pembrolizumab outperformed second-line chemotherapy with respect to the objective response rate (ORR) and the duration of response in recurrent locally advanced or metastatic EC, and in subgroup analysis revealed that the benefits were stronger in the Asian population than in other patients. As the first large, multicentre, randomized phase III study to compare the efficacy of immunotherapy plus chemotherapy and chemotherapy as first-line treatment for unresectable, locally advanced or metastatic EC or Siewert type I gastroesophageal junction cancer (GEJC), the KEYNOTE-590 (7) study demonstrated that pembrolizumab plus chemotherapy could provide significant and clinically meaningful improvements in overall survival, progression-free survival, and objective response rates compared with placebo plus chemotherapy in patients. At present, pembrolizumab is the only immunotherapeutic drug approved by both the American Food and Drug Administration (FDA) and National Medical Products Administration (NMPA) of China, and it has been recommended by both the NCCN guideline and CSCO guideline for the first-line treatment of advanced ESCC. Based on the good efficacy and safety of pembrolizumab, its application and exploration in neoadjuvant therapy for locally advanced ESCC have attracted great attention.

In theory, chemotherapy activates the immune system by activating antigens, which may improve the response of immunotherapies, enhance their efficiency, and reduce adverse effects (8). We previously conducted a single-arm, single-centre, phase II trial (NCT04389177) of pembrolizumab combined with paclitaxel and cisplatin as a neoadjuvant treatment for locally advanced ESCC (Keystone-001), and the interim analysis results confirmed the good efficacy and acceptable toxicity of pembrolizumab (9). Additional prospective phase II studies have also shown promising efficacy of the preoperative addition of immunotherapy to neoCT (10–12). However, it is unclear whether immunotherapy combined with neoCT could be more beneficial than the standard treatment (neoCRT). To the best of our knowledge, no randomized studies have been conducted to compare pembrolizumab combined with neoCT versus neoCRT in patients with locally advanced resectable ESCC.

Thus, we developed this multicentre, prospective, randomized-controlled, phase III clinical trial (Keystone-002) to assess the potential benefits of pembrolizumab combined with neoCT followed by oesophagectomy.

## Objective

This multicentre, prospective, randomized controlled, phase III clinical study aims at comparing the efficacy of pembrolizumab combined with neoCT versus neoCRT plus surgery in patients with locally advanced ESCC.

## Methods

### Study Design

**Figure 1:**
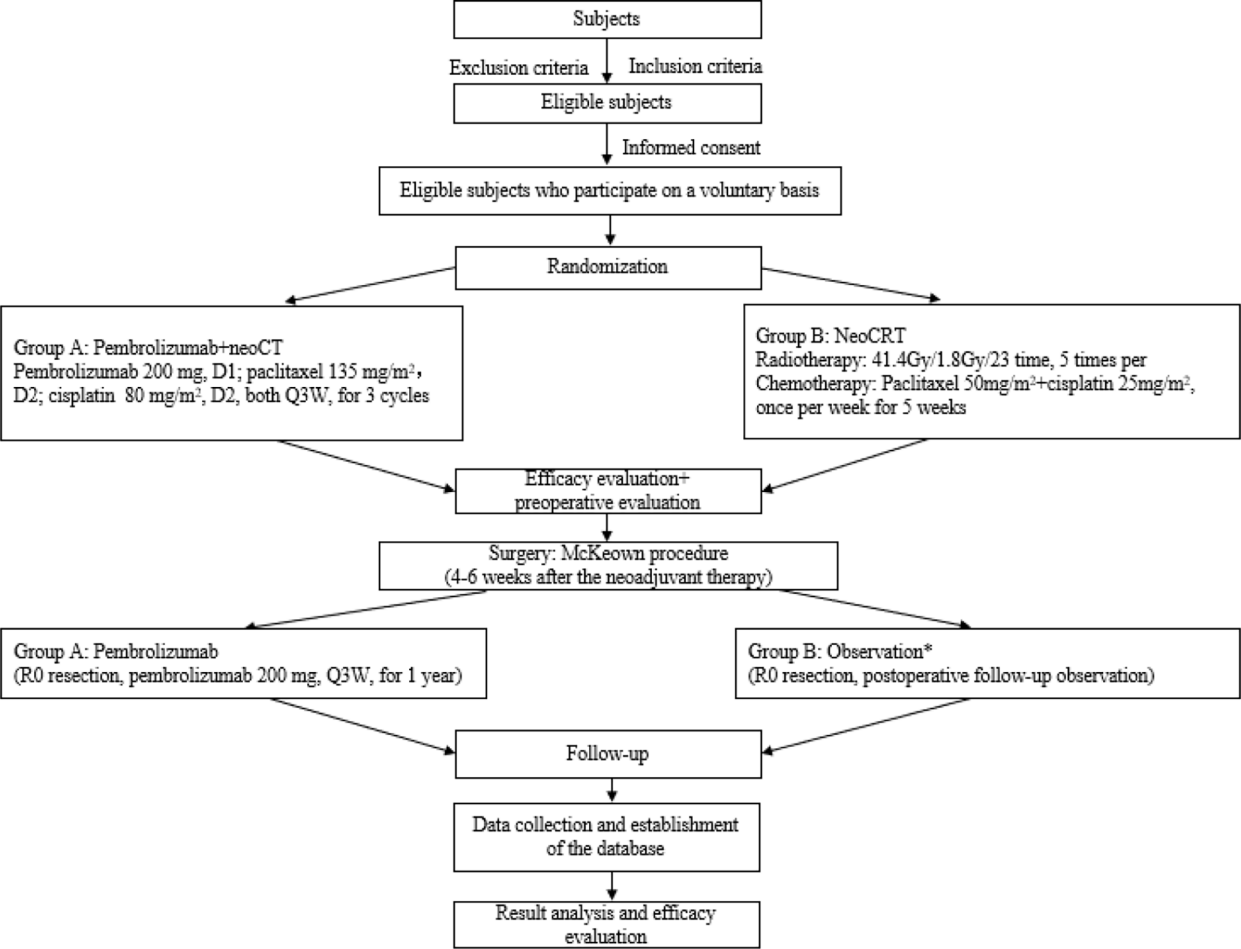
Study flowchart. *Considering the long enrolment time, the postoperative treatment in the neoCRT group might be adjusted according to the updated guidelines.

This is a multicentre, prospective, randomized controlled phase III clinical study, and locally advanced thoracic ESCC patients with cT1N2M0 or cT2-3N0-2M0 (high-risk lesions in T2N0M0) were enrolled to explore the effects of pembrolizumab plus chemotherapy. It is expected that 17 medical centres in China will participate, of which 6 medical centres have been approved by the local hospital ethics committee (Tianjin Medical University Cancer Institute and Hospital, Cancer Hospital of Chinese Academy of Medical Sciences, Ruijin Hospital Affiliated to Shanghai Jiaotong University, Liaoning Cancer Hospital, Shandong Province Hospital, and Tianjin Medical University General Hospital). The flowchart of this trial is presented in **Figure 1**. A central randomization system is used, with an allocation ratio of 2:1 for group A and group B, respectively.

**Figure 2:**
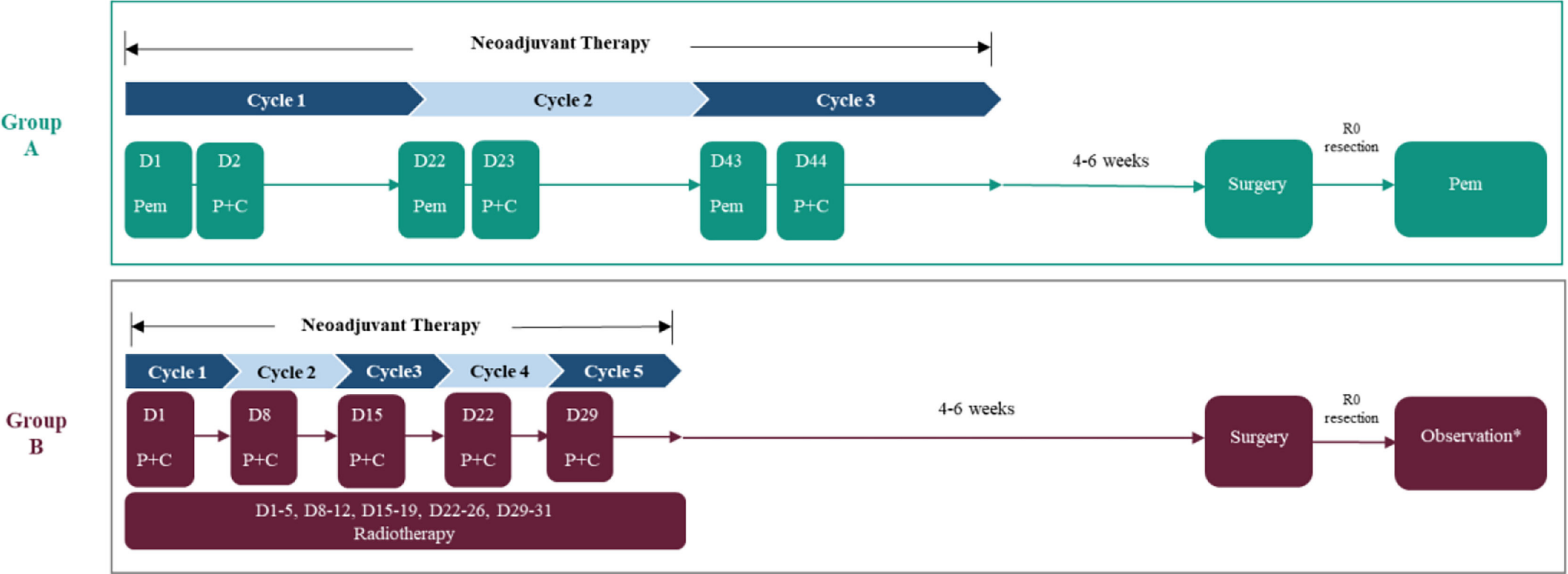
The therapeutic strategy for enrolled patients. Pem, pembrolizumab; P, paclitaxel; C, cisplatin. *Considering the long enrolment time, the postoperative treatment in the neoCRT group might be adjusted according to the updated guidelines.

The experimental group (group A) will receive pembrolizumab (200 mg intravenous (IV) D1 Q3W for 3 cycles), cisplatin (80 mg/m^2^ IV D2 Q3W for 3 cycles), and paclitaxel (135 mg/m^2^ IV D2 Q3W for 3 cycles) as neoadjuvant therapy, while the control group (group B) will receive cisplatin (25mg/m^2^ IV D1 Q1W for 5 cycles) and paclitaxel (50 mg/m^2^ IV D1 Q1W for 5 cycles) combined with radiotherapy (41.4 Gy, 1.8 Gy × 23 times, 5 times per week for 5 weeks consecutively). The McKeown procedure will be performed in both groups at 4–6 weeks (**Figure 2**). In addition, group A will also receive pembrolizumab alone as adjuvant therapy after surgery. The therapeutic session of group A will last from the first dose of pembrolizumab (day 1) to the last dose of the 1-year maintenance treatment after surgery or until the radiographically confirmed PD or other condition indicated for premature termination is observed. Group B will undergo a follow-up observation if R0 resection is achieved; however, considering the long enrolment time, the postoperative treatment might be adjusted according to the updated guidelines.

### Study Population

Patients with locally advanced ESCC will be eligible for the trial. The inclusion and exclusion criteria have been set by the investigators.

### Inclusion Criteria

1. Subjects need to sign an informed consent form;
2. Aged 18–75 years old, male or female;
3. Pathologically and radiographically confirmed as treatment-naive, locally advanced ESCC patients; staging: cT1N2M0 or cT2-3N0-2M0 (stage II/III, high-risk lesions in T2N0M0), the Eighth Edition (2016) of the TNM Staging Manual for Esophageal Squamous Cell Carcinoma developed by the American Joint Committee (AJCC);
4. Patients eligible for R0 surgical resection before treatment;
5. No suspicious lymph node metastases upon cervical colour Doppler;
6. Eastern Cooperative Oncology Group Performance Status (ECOG PS) score: 0–1;
7. The important functions will satisfy the following requirements (excluding subjects who receive a transfusion of any blood components and cell growth factors within 14 days): normal bone marrow reserve, neutrophil count ≥1,500/mm^3^, platelet count ≥100,000/mm^3^, haemoglobin level ≥5.6 mmol/l (9 g/dl); normal kidney function or serum creatinine ≤1.5 mg/day and/or creatinine clearance ≥60 ml/min; normal liver function or bilirubin level ≤1.5× ULN (upper limit of normal value, ULN), AST (aspartate transaminase, AST), and ALT (alanine transaminase, ALT) ≤1.5 × ULN;
8. Female patients of child-bearing age who have not received sterilization surgery must use a medically approved contraceptive agent during the study period or within 3 months after the treatment (e.g., intrauterine contraceptive device, contraceptive agents, or Condon); those who have not received sterilization surgery must have a negative serum or urine human chorionic gonadotropin (HCG) test within 7 days before the enrolment; and female patients must not be in the lactation period. Male patients of child-bearing age who have not received sterilization surgery must consent to the use of one medically approved contraceptive measure for themselves and their spouses during the study period and for 3 months posttreatment;
9. Subjects must agree to voluntarily participate in clinical research, have good compliance, and cooperate with safety and survival follow-up.

### Exclusion Criteria

Subjects who met any of the following criteria will be excluded.

1. History of radiotherapy, chemotherapy, hormone therapy, surgery, or molecular targeted therapy;
2. History of malignancies or concurrent malignancies, including carcinoma *in situ* or tumour history of more than 5 years (such as thyroid cancer);
3. History of other anti-PD-1/programmed death ligand 1 (PD-L1) treatments; a history of known allergies to macromolecular protein preparations or any component of PD-1;
4. Any active autoimmune diseases or any history of autoimmune diseases (including, but not limited to, autoimmune hepatitis, interstitial pneumonia, uveitis, enteritis, hepatitis, hypophysitis, vasculitis, nephritis, hyperthyroidism, and hypothyroidism; for patients with vitiligo or completely resolved childhood asthma, those not indicated for treatment in adulthood can be enrolled, and those indicated for bronchodilators for asthma will be excluded);
5. Currently receiving immunosuppressives or systemic or local hormone therapy for immunosuppression (prednisone at a dose >10 mg per day or other equivalent hormones) and continuing the treatment within 2 weeks before enrolment;
6. Symptomatic ascites or pleural effusion indicated for therapeutic puncture or drainage;
7. Poorly controlled heart symptoms or heart diseases, including (1) heart failure above New York Heart Association (NYHA) class II; (2) unstable angina pectoris; (3) a previous episode of myocardial infarction within 1 year; and (4) clinically significant supraventricular or ventricular arrhythmia indicated for treatment or intervention;
8. Coagulation disorders (prothrombin time (PT) >16s, activated partial thrombin time (APTT) >43s, thrombin time (TT) >21s, plasma fibrinogen (Fbg) >2g/l), haemorrhagic tendency, or currently receiving thrombolysis or anticoagulant therapy;
9. History of digestive tract conditions, such as oesophageal varix, active gastric and duodenal ulcer, ulcerative colitis, portal hypertension, active bleeding of the unresected tumour, or other conditions that may cause gastrointestinal bleeding and perforation as judged by the investigator;
10. History of severe haemorrhage or current severe haemorrhage (bleeding volume >30 ml within 3 months), haemoptysis (loss of fresh blood >5 ml in the past 4 weeks), or thromboembolic events within 12 months (including stroke events and/or transient ischaemic attack);
11. Active infection or fever >38.5°C for an unknown reason during the screening period or before the first dose (patients with tumour-related fever may be judged eligible by the investigator);
12. Experienced abdominal fistula, gastrointestinal perforation, or peritoneal abscess within 4 weeks before the first dose;
13. History or evidence of radiation pneumonitis, pneumoconiosis, interstitial pneumonia, pulmonary fibrosis, drug-related pneumonia, and severely impaired lung function;
14. Congenital or acquired immune deficiencies, such as human immunodeficiency virus (HIV) infection or active hepatitis (for those with aminotransferase levels not meeting the standards: hepatitis B (HBV): DNA ≥104/ml; hepatitis C (HCV): RNA ≥103/ml); chronic hepatitis B virus carriers: HBV DNA ≥2,000 IU/ml (≥104 copies/ml). The diagnostic criteria for active HBV infection include the following: (1) fatigue and digestive tract symptoms with no alternative explanations, including yellow urine, yellow eyes, and skin jaundice; (2) abnormal liver biochemical examination, mainly increased serum ALT and AST; patients with increased serum bilirubin may be included; (3) hepatitis B surface antigen (HBsAg) positive; (4) clear evidence that the serum HBsAg has been tested negative within 6 months; (5) hepatitis B core IgM antibody (anti-HBc IgM) positive 1:1,000 or more; (6) liver histology is consistent with changes in acute viral hepatitis; (7) serum HBsAg negative conversion, anti-HB-positive conversion during convalescence. The criteria for diagnosing chronic HBV infection were as follows: (1) HBsAg positive for more than 6 months after acute HBV infection or HBsAg positive for >6 months; (2) the duration of HBsAg positivity is unknown, and the patient is negative for anti-HBc IgM; (3) signs and symptoms of chronic liver disease, liver palms, spider nevus, hepatosplenomegaly, etc.; (4) repeated or persistent elevated levels of serum ALT, decreased levels of plasma albumin and/or increased levels of globulin, increased levels of bilirubin, etc.; (5) the liver pathology is consistent with the characteristics of chronic viral hepatitis; (6) serum HBeAg is positive or HBV DNA can be detected, and other causes of serum ALT elevation are excluded. During the study period, these subjects are not eligible for enrolment unless they receive antiviral treatment concomitantly;
15. Currently participating in other clinical trials or previously participated in another clinical trial within 1 month of the current study; indicated for other systemic antitumour treatments during the study period;
16. Received live vaccines within 4 weeks of the current study or indicated for live vaccines during the study period;
17. Known history of psychotropic substance abuse, alcohol abuse, or drug abuse;
18. Unable or unwilling to pay for the examinations and treatments not funded by the study (except for those arising from serious adverse events (SAEs) related to the investigational drug and combined radiotherapy and chemotherapy);
19. The subjects have, as judged by the investigator, other non-medical conditions that may lead to involuntary withdrawal from the study, such as family or social factors, conditions that may impair the safety of the subjects, or data and sample collection.

### Withdrawal Criteria

1. The subjects or their legal representatives ask for premature withdrawal;
2. Unexpected rapid disease progression, or radiographically confirmed disease progression;
3. The subjects face potential harms to their health if their participation continues;
4. Occurrence of any of the following conditions during neoadjuvant therapy: grade 4 haematological toxicity and grade 3 neutropenia with fever (>38.5°C) that do not return to normal after dose adjustment, or grade 3 thrombocytopenia with haemorrhage and haemorrhagic tendency, oesophageal perforation, concurrent severe pulmonary/mediastinal infection, haemorrhage, myocardial infarction, heart failure, and severe arrhythmia;
5. Occurrence of intolerable toxicities, including immune-related pneumonitis ≥grade 2, immune-related hepatitis ≥grade 3, transfusion reaction, or other immune-related toxicities;
6. Intolerant to surgery after neoadjuvant therapy; any delay of treatment for 2 weeks for any reason (including chemotherapy and immunotherapy);
7. Pregnancy or loss to follow-up;
8. Necessary withdrawal as judged by the investigator (the reasons for quitting should be recorded in detail, such as protocol violation and new complications);
9. All patients who withdraw prematurely will be followed up as per the protocol. The follow-up results will be recorded unless the subjects rescind their consent, refuse follow-up, or ask for a withdrawal.

### Sample Size Considerations

The sample size was calculated assuming that the median EFS is 20 months for neoCRT vs. 30 months for pembrolizumab combined with neoCT. A sample of 342 patients, including 228 in pembrolizumab combined with the neoCT group and 114 in the neoCRT group (2:1), was planned because 207 events are necessary to obtain a power of 80% to detect a hazard ratio of 0.67 with a two-sided significance level of 5% for the primary analysis and a 10% dropout rate. Enrolment will take approximately 2 years, and observation will take 2 years. One set of interim analyses will be performed when approximately 70% (145 EFS events) of the planned EFS events have occurred, and a Lan–DeMets error spending functions with the O’Brien–Fleming boundary will be used. The first interim analysis of overall survival was planned to occur if the interim analysis of EFS crossed the prespecified boundary. Two additional overall survival analyses are planned: an interim analysis at the time of the final EFS analysis and a final analysis when approximately 207 events occurred. The overall type I error will be controlled at 0.05 for EFS, and the overall survival will be controlled using the gate-keeping strategy.

### Randomization

Randomization will be performed using a central randomization system designed in advance. Considering the unfeasibility in clinical practice, neither the patients nor the surgeons will be blinded.

### Medication Treatment

The medical treatment details are shown in **Table 1**.

**Table 1:** Medical treatment of the patients.

Treatment group	Drug	Dose	Administration route	Treatment day	Cycle number
Experimental group (group A)	Pembrolizumab	200 mg	IV	Day 1, Q3W	3 + 17*^a^*
	Paclitaxel	135 mg/m^2^	IV	Day 2, Q3W	3
	Cisplatin	80 mg/m^2^	IV	Day 2, Q3W	3
Control group (group B)	Paclitaxel	50 mg/m^2^	IV	Day 1, Q1W	5
	Cisplatin	25 mg/m^2^	IV	Day 1, Q1W	5

^a^Pembrolizumab: 200 mg IV Q3W, day 1 of each cycle for 3 cycles, for neoadjuvant therapy; 200 mg IV Q3W, from the first dose of pembrolizumab (day 1) after surgery to the last dose of the 1-year maintenance treatment or until the radiographically confirmed PD or other condition indicated for premature termination, for adjuvant therapy.

### Radiotherapy

Among those receiving neoCRT, the total radiation dose will be split into 23 smaller doses of 1.8 Gy (a total of 41.4 Gy), and patients will receive cisplatin (25 mg/m^2^ intravenous (IV) D1 Q1W for 5 cycles) and paclitaxel (50 mg/m^2^ IV D1 Q1W for 5 cycles). Considering the long enrolment time, the postoperative treatment in the neoCRT group might be adjusted according to the updated guidelines.

### Surgery

1. Surgical timing: surgery will occur within 4–6 weeks after the end of the neoCRT and after the white cell blood count, platelet count, and liver and kidney functions returned to normal.
2. Surgical standard: the surgeries will be performed by thoracic surgeons with rich experience. The differences in surgical skills across the centres will be minimized through communications on the site of surgery and video reviews.
3. Surgical methods:

- The McKeown procedure can be performed using the da Vinci surgical robot, thoracoscope, or laparoscope, or by using an open approach, as judged appropriate by the surgeon.
- The surgery covered the steps below: the oesophagus will be dissociated through the right thoracic incision. The stomach will be separated through an abdominal incision, and a tubular stomach will be made. Oesophagogastric anastomosis will be performed on the left neck, followed by thoracic and abdominal lymph node dissection. Intraoperative macroscopic observation and postoperative pathological examination of surgical margins will be used to determine whether radical resection is achieved. An indwelling nasojejunal feeding tube or feeding tube through jejunostomy is recommended.
- Lymph node dissection: thoracic lymph node dissection covers the following areas: thoracic paraesophagus, bilateral recurrent laryngeal nerves, subcarina, bilateral peribronchial, and thoracic duct suspected of tumour invasion. Abdominal lymph node dissection covers the following areas: the superior border of the pancreas, diaphragmatic hiatus, left splenogastric ligament, and right hepatogastric ligament.
- Grouping of the lymph nodes: upper thoracic oesophagus (105), middle thoracic oesophagus (108), lower thoracic oesophagus (110), right recurrent laryngeal nerve (106recR), left recurrent laryngeal nerve(106recL), subcarina (107), right tracheobronchus (106tbR), left tracheobronchus (106tbL), supraphrenic (110), right cardia (1), left cardia (2), lesser curvature of the stomach (3), and left gastric artery (7). There should be at least dissected lymph nodes. The dissected lymph nodes must include the lymph nodes adjacent to the left and right recurrent laryngeal nerves. The bilateral recurrent laryngeal nerves should be clearly exposed. The dissection will be performed in accordance with the Japanese Classification of Esophageal Cancer, Eleventh Edition (13).

### Pathology Report

The pathology reports will include the following information: infiltration depth of the primary lesions, histological type, pathological status of the superior and posterior resection margins, circumferential margin, and lymph node involvement. There should be at least 15 lymph nodes submitted for pathological examination for each patient. Postoperative pathological assessment will be performed in accordance with the CROSS trial. The tumour regression grade (TRG) (14) and ypTNM staging will be assessed.

### Sample Processing

Tissue samples collected before neoadjuvant therapy and those collected intraoperatively (for all cases): the primary lesions, paracancerous lesions (within 2 cm from the primary lesions), and normal oesophageal tissues (beyond 2 cm from the primary lesions) will be collected in duplicate. One sample will be loaded into a common cryopreservation tube, and the other will be loaded into an EP tube containing the RNA preservation solution. Both samples will be preserved at -80°C. Before neoadjuvant therapy, at 3 weeks after the end of chemotherapy or before surgery, 5 ml of peripheral blood samples will be collected from each patient. The blood samples will be centrifuged at 3,000 r/min for 10 min within 2 h after collection. The serum, plasma, and white blood cell layers will be collected and loaded into a 1- or 2-ml cryopreservation tube and placed at -80°C.

## Study Endpoints

### Primary Endpoints

The primary endpoint is event-free survival (EFS). EFS is defined as the time from randomization to any of the following events: contraindicated for surgery due to disease progression, postoperative local relapse, distant metastasis, or death.

### Secondary Endpoints

1. The 1-, 3-, and 5-year OS. OS is defined as the time from randomization to the patient death.
2. The 1-, 3-, and 5-year disease-free survival (DFS). DFS is defined as the time from the date of surgery to the day of tumour recurrence, tumour progression, or patient death.
3. Short-term outcomes include the major pathological response (MPR) rate, pathological complete response (pCR) rate, objective response rate (ORR), R0 resection rate, and 30- and 90-day perioperative complication rates. ORR was defined as the percentage of subjects in the analysis population who had a complete response (CR: disappearance of all target lesions) or a partial response (PR: ≥30% decrease in the sum of diameters of target lesions) per Response Evaluation Criteria in Solid Tumors version 1.1 (RECIST 1.1), as assessed by the investigator.
4. Quality of life (European Organization for Research and Treatment of Cancer quality of life, EORTC QLQ)-C30, EORTC QLQ-OES18) and Patient-Generated Subjective Global Assessment (PG-SGA) (15) scores of patients with stage II/III thoracic ESCC were compared.

• EORTC QLQ-C30 and EORTC QLQ-OES18:

The QLQ-C30 scale includes five functional scales (physical, role, cognitive, emotional, and social), three symptom scales (fatigue, pain, and nausea and vomiting), six single items (dyspnoea, sleep disorder, loss of appetite, constipation, diarrhoea, and economic impact), and a global health status/QoL scale (16).

The EORTC QLQ-OES18 is a disease-specific questionnaire for assessing the quality of life of patients with oesophageal cancer and is often used along with the QLQ-C30 as a disease-specific treatment indicator. The EORTC QLQ-OES18 assesses 18 symptoms, including dysphagia, pain, reflux, eating, swallowing saliva, choking, dry mouth, taste, cough, and speech (17).

• PG-SGA score:

The PG-SGA score is a subjective assessment of the overall nutritional status of patients and includes two parts: patient self-assessment and medical staff assessment. The patient self-assessment is further divided into four dimensions: body weight, food intake, symptoms, and activity and physical function. The medical staff assessment includes three dimensions: disease and its relation to nutritional requirements, metabolic demands, and physical exam.

## Exploratory Endpoints

The biological prediction model of sensitivity to immunotherapy combined with neoCT and neoCRT will be built for ESCC based on biological detection and bioinformatics analysis. Molecular markers for sensitivity to neoadjuvant therapy will be identified. The relationship between biomarkers in tumour tissues/blood and efficacy will be evaluated.

1. Sample collection and storage: blood samples will be collected from patients before and after neoadjuvant therapy. The serum and whole blood samples will be prepared for ELISA and flow cytometry. Tumour tissues will be collected by endoscopic biopsy before and after neoadjuvant therapy and cryopreserved in the tissue bank.
2. Blood sample collection: changes in the serum levels of cytokines will be detected by enzyme-linked immunosorbent assay (ELISA) at the protein level. Changes in the levels of immune cells (lymphocytes, natural killer (NK) cells, and other tumour immunity-related cell populations) will be detected by flow cytometry in blood samples.
3. Tissue sample detection: biopsy samples will be subjected to second-generation sequencing after preprocessing. The detection of transcriptome gene-level and exon-level expression changes will be prioritized.
4. Building a sensitivity prediction model: important indicators will be extracted based on clinical data, haematological tests, second-generation sequencing results, and bioinformatic analysis. Then, the biological prediction model will be built. We are also concerned with the discovery of driver genes in oesophageal cancer.

## Safety Evaluation

### Adverse Events

1. Definition of adverse events (AE): AE is any adverse medical event in a patient or subject administered a pharmaceutical product that does not necessarily have a causal relationship with the treatment.
2. AE recording and reporting
  a. The course, severity, onset time, duration, management, and outcomes of AEs during the trial will be recorded in detail.
3. AE grading
  a. The severity of AEs will be divided into grades 0–4 according to the National Cancer Institute Common Toxicity Criteria (NCI-CTCAE5.0).
4. Relationship between AEs and the study treatment

The relationship between AEs and the study treatment is divided into five levels: definitely relevant, probably relevant, possibly relevant, possibly irrelevant, and definitely irrelevant.

### Serious Adverse Events

If serious adverse events (SAEs) occur during the trial, the subjects will immediately discontinue the treatment. The investigator will take appropriate protective measures and provide symptomatic treatment at once. The occurrence of SAEs should be reported to the principal investigator within 24 h. The investigator shall fill in the SAE Report Form and sign with a date.

### Immune-Related Adverse Events

Immune-related adverse events (irAEs) are clinically significant adverse reactions related to the immune mechanisms of the drug.

### Efficacy Evaluation

Efficacy endpoints, including ORR, will be estimated along with their 95% confidence interval (CI). OS and median survival will be estimated using the Kaplan–Meier method along with their 95% CI.

### Safety Analysis

AEs are summarized in the form of frequencies and percentages. The results of laboratory tests (routine blood test, blood biochemistry), ECOG, vital signs, and electrocardiogram (ECG) will be described.

### Statistical Analysis

Statistical analyses will be performed using Statistical Package for the Social Sciences (SPSS) version 27.0 software. Survival will be estimated by Kaplan–Meier methods and analysed using the log-rank test. Student’s t-test and analysis of variance (ANOVA) will be conducted for the comparison of continuous variables, while the χ^2^ test or Fisher’s exact test will be used to compare categorical variables. p < 0.05 will be set as the significance level.

### Current Status

The trial has been approved by the ethics committees of 6 medical centres in China. Recruitment of patients started in December 2021. Recruitment is still ongoing, and 342 patients will be recruited by December 2023.

## Discussion

The CROSS trial (4) and the NEOCRTEC5010 trial (5) confirmed the effective benefits of neoCRT in locally advanced EC. However, in the CROSS trial (4), the majority of participants were oesophageal adenocarcinoma (EAC) patients. The NEOCRTEC5010 trial was a phase III randomized trial of patients with ESCC in China. Patients were divided into a neoCRT+surgery group and a surgery-alone group, with a pCR rate up to 43.2% in the neoCRT group and a median survival of 100.1 months in the neoCRT plus surgery group versus 41.7 months in the surgery-alone group (p < 0.001). Based on the findings above, neoCRT plus surgery has become a recommended option for locally advanced ESCC. However, the improved pCR rate in neoCRT did not provide a more significant long-term survival advantage than in neoCT (18, 19). In addition, the clinical application of neoCRT is restricted due to the toxicity of CRT and the superimposed toxicity of chemotherapy and radiotherapy. Heavy toxicity may lead to severe acute adverse reactions during chemoradiotherapy; thus, the dose of chemotherapy or radiotherapy would be decreased, which to a large extent weakens the patients’ treatment adherence. In addition, neoCRT may further add to the difficulty of surgical procedures (e.g., tissue adhesion and oedema) and increase perioperative complications (e.g., respiratory insufficiency or even failure due to radiation pneumonitis) or even the perioperative mortality soaring, which undesirably counteracts the survival benefits expected from neoCRT. All these problems have restricted the clinical use of concurrent chemoradiotherapy before surgery.

Taken together, an ideal neoadjuvant therapy is one that achieves a higher pCR rate and long-term survival rate with minimal surgical disturbance and ease of clinical use. With immunotherapy recommended for first-line and second-line treatment in advanced or metastatic EC and increasingly applied in the clinic, which is based on its long-term survival benefits and tolerable safety, the novel combination based on immunotherapy and chemotherapy as neoadjuvant treatment for locally advanced EC is of great concern and anticipated. Preclinical studies have proven that chemotherapy can activate the human immune system, thereby enhancing the role of immune checkpoint inhibitors (8, 20). The action of neoCT and immune checkpoint inhibitors is not simply superimposed. The two treatment modalities may also work synergistically.

We conducted a phase II study (NCT04389177) on the safety and efficacy of pembrolizumab combined with paclitaxel and cisplatin as a neoadjuvant treatment for locally advanced resectable ESCC (Keystone-001). The interim analysis (9) was presented at the European Society for Medical Oncology (ESMO) Immuno-Oncology congress 2021, and all patient recruitment has been closed. The interim results confirmed that the treatment of pembrolizumab combined with paclitaxel and cisplatin was safe (no adverse events related to neoadjuvant therapy of grade 4 or higher) and did not cause a delay in surgery. The R0 resection rate was 100%, with an MPR of 21/29 (72.4%), pCR of12/29 (41.4%), and ORR of 28/29 (96.6%). Pembrolizumab combined with paclitaxel and cisplatin as a neoadjuvant therapy was associated with few side effects, did not delay surgery, and induced a high major pathological response. This treatment markedly lowered the difficulty of surgical resection compared with neoCRT, and the patients generally achieved good postoperative recovery. The findings of the studies above will be further analysed and submitted to journals. The phase II NICE study (10) enrolled 60 patients with locally advanced ESCC who received neoadjuvant treatment with immunotherapy plus chemotherapy before surgery; pCR (ypT0N0) was identified in 20 (42.5%) of 47 patients, and 5 (10.6%) patients had a complete pathological response of the primary tumour but residual disease in lymph nodes alone (ypT0N+). The grade 3–5 treatment-related adverse event (TRAE) rate was 53.3%, and the TRAE-induced discontinuation rate was 6.7%. Similarly, the NIC-ESCC2019 study (11) was also designed as a single-arm, phase II study to explore the efficacy of immunotherapy plus chemotherapy as neoadjuvant therapy for resectable, locally advanced ESCC. The results showed that pCR was achieved in 35.3% of patients and that 23.5% of patients had MPR. The ORR was 66.7%, and the in-hospital mortality rate was 0%. Grade 3 TRAEs only occurred in 11% of patients, and there were no grade 4 or 5 TRAEs.

On the basis of previously reported evidence and clinical experiences, the addition of immunotherapy to neoCT in ESCC demonstrated promising efficacy with acceptable toxicity, thereby supporting further investigation in a randomized phase III clinical trial. Thus, we designed this multicentre, prospective, randomized-controlled, phase III clinical study to compare the addition of pembrolizumab plus neoCT versus neoCRT. According to our hypothesis, pembrolizumab combined with neoCT will result in a better oncological outcome and long-term quality of life for patients with locally advanced ESCC.

## Data Availability Statement

The raw data supporting the conclusions of this article will be made available by the authors, without undue reservation.

## Ethics Statement

The studies involving human participants were reviewed and approved by the Ethics Committee of Tianjin Medical University Cancer Institute and Hospital (No. E20210314). The patients/participants provided their written informed consent to participate in this study.

## Author Contributions

XS, HJ, and YL contributed to the study design of this trial as well as writing. WZ and QP contributed to the study design of the radiation part. GZ and BM contributed to the study design of the pathologic part. FL contributed to the calculation of the sample size. WZ, LL, and XR contributed to the study design of the immunochemotherapy part. CZ, JY, XD, ZM, and CC contributed to the study design of surgery. PZ, YM, LZ, HL, and XK contributed to the editing of the manuscript. All authors approved the final version of the manuscript. All authors contributed to the article and approved the submitted version.

## Funding

This trial was partially funded by the Beijing Xisike Clinical Oncology Research Foundation (No. Y-MSD2020-0346, and No. Y-MSDZD-2021-0239).

## Conflict of Interest

The authors declare that the research was conducted in the absence of any commercial or financial relationships that could be construed as a potential conflict of interest.

## Publisher’s Note

All claims expressed in this article are solely those of the authors and do not necessarily represent those of their affiliated organizations, or those of the publisher, the editors and the reviewers. Any product that may be evaluated in this article, or claim that may be made by its manufacturer, is not guaranteed or endorsed by the publisher.

## Abbreviations

AEs, adverse events; CSCO, Chinese Society of Clinical Oncology; cCR, complete clinical response; DFS, disease-free survival; EC, oesophageal cancer; EFS, event-free survival; EFS, oesophageal squamous cell cancer; FDA, American Food and Drug Administration, irAEs, immune-related adverse events; MPR, major pathological response rate; NCCN, National Comprehensive Cancer Network; neoCT, neoadjuvant chemotherapy; neoCRT, neoadjuvant chemoradiotherapy; NMPA, National Medical Products Administration; ORR, objective response rate; OS, overall survival; pCR, pathological complete response rate; PFS, progression-free survival; QOL, quality of life; SAEs, serious adverse events; TRAE, treatment-related adverse events; TRG, tumour regression grade.
